# Mesenchymal Stem Cells as a Cornerstone in a Galaxy of Intercellular Signals: Basis for a New Era of Medicine

**DOI:** 10.3390/ijms22073576

**Published:** 2021-03-30

**Authors:** Silvia Fernández-Francos, Noemi Eiro, Luis A. Costa, Sara Escudero-Cernuda, María Luisa Fernández-Sánchez, Francisco J. Vizoso

**Affiliations:** 1 Research Unit, Fundación Hospital de Jove, 33290 Gijón, Spain; silviafernandezfrancos@gmail.com (S.F.-F.); luiscostaperez@gmail.com (L.A.C.); 2 Department of Physical and Analytical Chemistry, Faculty of Chemistry, University of Oviedo, 33006 Oviedo, Spain; saraescuderoc33@gmail.com (S.E.-C.); marisafs@uniovi.es (M.L.F.-S.)

**Keywords:** secretome, extracellular vesicles, exosomes, MSC *in vitro* production, MSC large-scale expansion, ex vivo MSC modifications, bioreactor

## Abstract

Around 40% of the population will suffer at some point in their life a disease involving tissue loss or an inflammatory or autoimmune process that cannot be satisfactorily controlled with current therapies. An alternative for these processes is represented by stem cells and, especially, mesenchymal stem cells (MSC). Numerous preclinical studies have shown MSC to have therapeutic effects in different clinical conditions, probably due to their mesodermal origin. Thereby, MSC appear to play a central role in the control of a galaxy of intercellular signals of anti-inflammatory, regenerative, angiogenic, anti-fibrotic, anti-oxidative stress effects of anti-apoptotic, anti-tumor, or anti-microbial type. This concept forces us to return to the origin of natural physiological processes as a starting point to understand the evolution of MSC therapy in the field of regenerative medicine. These biological effects, demonstrated in countless preclinical studies, justify their first clinical applications, and draw a horizon of new therapeutic strategies. However, several limitations of MSC as cell therapy are recognized, such as safety issues, handling difficulties for therapeutic purposes, and high economic cost. For these reasons, there is an ongoing tendency to consider the use of MSC-derived secretome products as a therapeutic tool, since they reproduce the effects of their parent cells. However, it will be necessary to resolve key aspects, such as the choice of the ideal type of MSC according to their origin for each therapeutic indication and the implementation of new standardized production strategies. Therefore, stem cell science based on an intelligently designed production of MSC and or their derivative products will be able to advance towards an innovative and more personalized medical biotechnology.

## 1. Introduction

Despite advances in medicine, there are still shortcomings in the treatment of many inflammatory, degenerative, or cancerous diseases, for which there is no curative treatment option. Among this wide range of pathologies, which will affect 40% of the population at some point in their life, those of immunological, degenerative, ischemic, or cancerous origin stand out. To this we must add that, at present, the social demand to find treatments for a gradually wide spectrum of rare diseases is increasing. However, to this cocktail of urgent needs, we must also add the problem of infections (especially due to the looming crisis of resistance of many bacterial strains to antibiotics), the emergence of new pandemics (such as that caused by the coronavirus), and the dramatic and progressive increase in average life expectancy throughout the world population. All this aggravates and extends the dilemma of effective therapeutic possibilities.

Faced with these realities, there is a growing exigency for new therapeutic alternatives that assume the real challenges presented by most of these “orphan diseases”—the control of the inflammatory process and tissue regeneration. For this, we count on the novel paradigms of science and medicine, which will contribute to glimpsing new solutions for old problems and for emerging challenges that will arise in the future. In this context, the development of studies around the archetype of stem cells and regenerative medicine represents one of the greatest endeavors in the history of medical science. Technological-biological advances have allowed us to raise great expectations on the basis of the different types of stem cells (embryonic stem cells, induced pluripotent stem cells (iPS) and adult stem cells, including hematopoietic stem cells, neural stem cells and mesenchymal stem cells (MSC)). Hope is particularly focused on MSC, observing an exponential increase in research based on them [1].

This is due to their influence in many basic aspects of cell biology, making a multi-faceted therapeutic approach possible and breaking the classic concept of the pharmaceutical industry of “one disease: a single therapeutic target” [2].

Under this broad angle, Caplan recently named these cells as “medicinal signaling cells” [3]. However, a perspective vision on the evolution of the investigations and potential applications of MSC in the last two decades can be noticed: (i) from their regenerative interest to their anti-inflammatory potential, and (ii) from cell therapy to the possibility of a cell-free therapy based on the products derived from its secretome. Moving on, we can also guess the need for future orientations of the paradigm: recognition of heterogeneity of MSC according to their tissue origin and donors, feasible and standardized production, and application of functional tests prior to therapeutic applications. Further, the possibility of influencing the achievement of biological products of MSC, suitable for the treatment of each specific pathological process, opens the doors to a pioneering way to address these critical health issues by improving the quality of life of patients.

## 2. MSC: Nomenclature, Properties, Heterogeneity, and Reality

From the mid-1960s, the soviet scientist Alexander Friedenstein demonstrated that mouse bone marrow and other blood-forming organs contained clonogenic progenitor cells that could give rise in culture to fibroblasts, as well as other mesodermal cells [4]. He observed that these precursor cells did not belong to the hematopoietic cell lineage and had the ability to generate bone and cartilage-forming cells. Nowadays, it is known that MSC appear, from the fetal/neonatal period to the stage of stromal tissue formation in the adult, in one seemingly ubiquitous localization in most vascularized tissues. They are fibroblast-like populations that are also known on different terms as “mesenchymal progenitor cells”, “mesenchymal adult stem cells”, “mesenchymal stromal cells”, “multipotential stromal cells”, “interstitial stem cells”, “marrow stromal cells”, or “medicinal signaling cells” [3]. The Mesenchymal and Tissue Stem Cell Committee of the International Society for Cellular Therapy established in 2006 the minimal identifying characteristics for human MSC: (a) plastic-adherent cells when maintained in standard culture conditions; (b) expression of CD105, CD73, and CD90, and lack expression of CD45, CD34, CD14, or CD11b, CD79a, or CD19 and HLA-DR surface molecules; and (c) capacity to differentiate into osteoblasts, adipocytes, and chondroblasts *in vitro* [5]. In addition, MSC are considered as immune-privileged cells since they do not express neither the major histocompatibility complex (MHC) II nor costimulatory molecules, such as CD86, CD40, or CD80, and also express a low level of MHC [6].

Bone marrow, subcutaneous fat, Wharton’s jelly, and umbilical cords are the more used sources of MSC [7] named BM-MSC, SF-MSC, WJ-MSC, and UC-MSC, respectively. However, others such as fetal/neonatal tissues [8,9,10,11], dental pulp [12], or placenta [8] are becoming of increased interest.

### 2.1. Biological Implication of the Mesodermical Origin from MSC

MSC seem to present a certain embryological imprint due to their mesodermal origin, which is translated, for example, in their plastic capacity to transform into adipose, osteoblastic, and chondroblastic cells. MSC are recognized for their fundamental role in the structural support of different tissue and organs. Notably, those of greater functional capacity, such as the immune system and the circulatory system, also derive from this embryological layer. Examples of specialized interstitial cells are the myofibroblasts, which regulate blood flow; interstitial cells of Cajal, which act as pacemakers to regulate smooth muscle cell contractility; or the synovial fibroblasts, which regulate the quality of synovial fluid [13,14]. The bone marrow (BM) and lymph nodes (LNs), intestinal Peyer’s patches, and the spleen are also examples of biological niches in which MSC play an essential role in regulating haematopoietic stem cell (HSC) activity through the expression of chemokines, cytokines, and lipid gradients, among others [15]. Furthermore, the evidence indicates that the biology of the mesoderm progresses through an expansive evolution in such a way that a part of the mesoderm evolves towards the constitution of a stroma intimately interconnected with the tissues that derive from the ectoderm or endoderm. In this context, MSC, whose functional heterogeneity seems to reflect the microenvironment derived from interactions with nearby cells, are in turn adapted to perform organ-specific functions [16,17]. Therefore, although MSC show a perivascular location in different anatomical locations, they differ in their functional capacity to generate paracrine signals. In fact, differences in the bioactive factors secreted by MSC depending on their location in the different organs and tissues have been described [18]. This idea breaks the hitherto widespread concept attributed to MSC of “one-cell-does-it-all”. In addition, the MSC heterogeneity must be taken into account when choosing the most appropriate type of MSC for each specific therapeutic application [2].

Thus, MSC participate in a “galaxy” of intercellular interactions as temporal–spatial regulators of tissue homeostasis. In fact, it is progressively being demonstrated that in many diseases with an inflammatory and degenerative base, there is a depletion or dysfunction of MSC, such as systemic lupus erythematosus (SLE), diabetes mellitus (DM), and rheumatoid arthritis (RA) [2].

### 2.2. MSC in the Context of a “Galaxy” of Intercellular Signals

**Figure 1:**
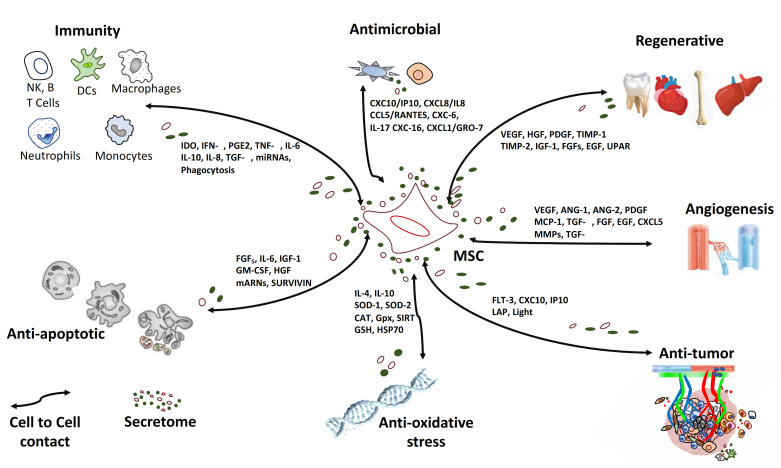
Mesenchymal stem cells (MSC) in the context of a “galaxy” of intercellular signals. Mesenchymal stem cells participate in different physiological processes through secreted factors (secretome) or by cell-to-cell contact.

Among the “galaxy” of intercellular signals in which MSC participate, those of an immunomodulatory, regenerative, anti-oxidative stress, angiogenic, anti-fibrotic, anti-tumoral, and anti-microbial nature draw attention (Figure 1).

#### 2.2.1. Anti-Inflammatory Effect

Inflammation is a protective response to harmful external stimuli and helps repair and remodel tissues, but when it is deregulated it can have detrimental effects [19]. MSC can exert pro-inflammatory and anti-inflammatory effects depending on the immune status of the microenvironment. The pro-inflammatory effect of MSC is beneficial throughout the early phases of inflammation, but its anti-inflammatory effects are useful during the later phases, when excessive immune activation would cause tissue damage or acute injury [20].

When tissue levels of inflammatory cytokines, such as IFN-γ and TNF-α, are low, MSC can accomplish pro-inflammatory actions. However, MSC are activated in the presence of elevated levels of these inflammatory cytokines and/or LPS. These latter conditions result in the upregulation of soluble anti-inflammatory factors, such as cyclooxygenase 2 (COX2), IDO, nitric oxide (NO), TGF-β1, PGE2, and HLA-G5 [21,22]. Consequently, and although the molecular mechanisms responsible have not been fully understood, MSC result in immunoregulatory effects on all of the immune cell types: (i) execute a suppressive effect on the proliferation of T cells (caused by cell cycle arrest in the G0/G1 phase rather than by the induction of T cell apoptosis) [23] and attenuate their functionality [24,25]; (ii) interact directly with B cells and can reduce plasmablast formation as well as to promote the induction of regulatory B cells (Bregs) [26]; (iii) polarize monocytes (M0) toward IL-10 producing anti-inflammatory M2 phenotype [27] and reprogram M1 macrophages to the M2 phenotype [28]; (iv) suppress natural killer (NK) cell proliferation, cytotoxicity, and cytokine secretion; and (v) inhibit the maturation, differentiation, and migration of dendritic cells (DCs), and reduce the cell-surface expressions of MHC-II molecules, CD11c, and CD83 [29].

#### 2.2.2. Regenerative Effect

Numerous studies indicate that the rate of MSC survival engraftment and the number of newly generated cells after MSC transplantation seems to be too low to explain the beneficial effects induced by MSC [30,31]. However, it is known that MSC secrete a wide range of biologically active factors such as growth factors, cytokines, active lipids, or extracellular microvesicles, which contribute decisively to tissue regeneration [32]. Thus, paracrine signaling of MSC has been suggested as the main mechanism of their regenerative action on the different cell types of both parenchymal and mesenchymal cells [33]. These mechanisms include the sequential process involving in tissue regeneration, such as migration, anti-inflammatory/immunomodulatory effect, accelerated re-epithelization, improved ECM production, and remodeling. Among the factors responsible for these mechanisms are inflammatory proteins (IL-1, -6, -8–11, -13; PGE2, MCP-1), growth factors (EGF, KGF, TGF-β, HGF, FGF, VEGF, GF-1, PDGF, BNDF, NGF-3, I G-CSF, GM-CSF, and PGE2), and ECM proteins (MMP-1, -2, -3, -7; TIPM-1 y 2, ICAM, collagens, laminin, elastin, and decorin). In addition, increased angiogenesis has been proposed as another one of the main mechanisms of regenerative effect from MSC by their paracrine action. An appropriate revitalization requires the formation of new blood vessels, which is a fundamental process for the delivery of oxygen, nutrients, and growth factors to the damaged tissues. MSC secrete molecular factors enhancing the proliferation and migration of endothelial cells, such as VEGF, PDGF, ANG-1 y 2, EGF, FGF, TGF-β1, TGF-α, MCP-1, CXCL5, and MMPs [34,35].

#### 2.2.3. Anti-Fibrotic Effect

A pro-fibrotic environment orchestrated by subjacent factors such those derived from oxidative stress, inflammation, and aging is present in many progressive and terminal maladies. This scenario implies the excessive deposition of ECM proteins, such as fibronectin, collagen I, and collagen III. As a result, biological functions are impaired, and the regeneration ability of tissues is diminished [36]. *In vivo* studies demonstrate an anti-fibrotic effect of MSC. Thus, for example, it was reported that WJ-MSC exert anti-fibrotic actions against skeletal muscle fibrosis, primarily via MMP-1 [37]. MSC also prevent renal or hepatic fibrosis via VEGF and HGF secretion, and further suppress TGF-β1-induced fibrotic changes [38,39], with TGF-β1/Smad pathway being an important pathogenic mechanism in tissue fibrosis [40,41]. The use of MSC secretome has been also shown to have anti-fibrotic effects by secretion of some growth factors and cytokines, such as HGF, TGF-β3, TNF-α, and IL-10 [17,42,43]. On the other hand, it has been indicated that BM-MSC-derived exosomes and other stem cell-derived secretomes can reduce liver fibrosis [17,44]. Recent data have also suggested that human umbilical cord mesenchymal stem cells (hUC-MSC)-derived exosomes could inhibit dermal fibroblast–myofibroblast transition by inhibiting the TGF-β1/Smad2/3 signaling pathway [45].

#### 2.2.4. Anti-Oxidative Stress Effect

Oxidative stress is concomitant with cell injury, inflammation, and dysregulated metabolism and, therefore, is a key pathophysiological mechanism of many diseases [46]. Oxidative stress refers to a deviation from the physiological redox state and an increase in pro-oxidants, or free radicals, that structurally change lipids, proteins, and DNA in a way that causes pathology or damage to a cell or tissue [47]. The most widely studied free radicals are reactive oxygen species (ROS), which can also include reactive molecules that have a stable charge. The three major endogenous ROS include the superoxide anion (O_2_ (−)), hydroxyl radical ((^.^)OH), and hydrogen peroxide (H_2_O_2_) [48,49]. The dynamic equilibrium of ROS production and metabolism is capital for the maintenance of the normal function of cells and tissues. If this balance is disrupted, it can lead to oxidative stress and a chain of tissue breakdown [50]. What is more, although the interactions between the immune system and oxidative stress are not fully understood, leukocytes and pro-inflammatory mediators intensify the formation of free radicals and perturb the redox environment creating a positive feedback cycle [51]. Neutrophils appear to be key mediators of oxidative stress in inflammation. These cells harbor an abundance activity of myeloperoxidase (MPO), a potent oxidant that plays an important role in oxidative stress by catalyzing H_2_O_2_ to hypochlorite [52,53].

Several studies have demonstrated that MSC are highly resistant to oxidative insult, which is associated with constitutively expressed antioxidant enzymes (SOD1, SOD2, catalase (CAT), glutathione peroxidase (Gpx) and high levels of the antioxidant glutathione (GSH)) [54]. In addition, MSC express heat-shock protein 70 (HSP70) and sirtuin (SIRT), which may also play a role in the resistance to oxidative stress [55]. Antioxidant effects of MSC therapy have been observed *in vitro* in a wide range of cell types (immune cells, endothelial cells, fibroblasts, cardiomyocytes, hepatocytes, renal cells, glial cells, neurons, pancreatic islet cells, and skeletal muscle cells) and *in vivo* on many disease models (aging, gastrointestinal inflammation, cognitive disorders, ischemic injuries, diabetic damages, septic insults, and chemotherapy- or radiation-induced harm to several organs). These studies revealed several mechanisms by which MSC have antioxidant effects, including direct scavenging of free radicals, promoting endogenous antioxidant defenses, immunomodulation via reactive oxygen species suppression, altering mitochondrial bioenergetics, and donating functional mitochondria to damaged cells [46]. On the other hand, MSC can also directly decrease ROS and MPO activity in stimulated monocytes and macrophages, which suppress their pro-inflammatory phenotype [56,57]. These data suggest that MSC not only suppress the immune system to prevent oxidative injury, but also that their mechanism of immunosuppression is reliant on their antioxidant properties.

#### 2.2.5. Anti-Apoptotic Effect

Apoptosis is a complex process that tightly regulates the rate of cell division and death and triggers a suicide program with DNA fragmentation enhancing, membrane of nucleus swelling, cytoplasm shrinking, and finally cell death [58]. Multiple studies have investigated the underlying antiapoptotic mechanisms from MSC in various organ injury models. Thus, for example, it was shown that human adipose-derived MSC (AD-MSC) are required for resisting apoptosis by upregulating the proliferation marker Ki67 and the antiapoptotic markers BCL2 and SURVIVIN, and by downregulating markers of apoptosis, TUNEL, annexin V, CASPASE3, and CASPASE9 [59]. It was also recently demonstrated that miR-146a-5p-riched BM-MSC exosomes reduce neuronal apoptosis and inflammation associated with the inhibition of microglial M1 polarization by downregulating the expression of IRAK1 and NFAT5 [60].

#### 2.2.6. Anti-Tumor Effect

The interactions between tumor cells and the non-malignant stromal cells exert a primary contribution in the pathophysiology of cancer. Emerging data indicate that one of the most heterogeneous effects from MSC according to their origins are these ones on tumors [61,62]. It is assumed that the pro- or anti-tumorigenic effect of MSC will depend on the origin of the MSC and the type of tumor. In this sense, the existing data seem to converge on the fact that MSC originated in the uterus and that pregnancy-related tissues have a broader antitumor effect, thus they could be good candidates for oncological therapies [63].

It has been shown that MSC secrete high amounts of cytokines, which induce inhibition of tumor growth *in vivo* in breast cancer cells, such as IFN-α, DKK-1/3, IL12, TRAIL, TNFSF14 (also known as LIGHT), FLT-3 ligand, CXCL10, and LAP [64,65,66,67]. It has also been reported that the anti-tumor effect of MSC may be partly related to the activity of the tissular inhibitors of matrix metalloproteinase TIMP-1 and TIMP-2, present in their secretome [64,68], with the inhibition of MMPs being associated with the suppression of migration and invasion of cancer cells.

Other anti-tumor mechanism of MSC may be via extracellular vesicles (EVs). Cancer cells have been shown to internalize a greater percentage of exosomes when compared to normal cells [69,70]. These EVs produced by MSC are potentially responsible for many of their antitumor effects. Thus, for example, EVs from human UC-MSC reverse the development of bladder carcinoma cells, possibly by downregulating the phosphorylation of Akt protein kinase and upregulating cleaved caspase-3 [71], with exosomal miRNA from AD-MSC suppressing the proliferation of ovarian cancer cells [72].

#### 2.2.7. Anti-Microbial Effect

It is relevant to consider that MSC are usually resistant to viral infection due to their expression of interferon (IFN), and, subsequently, IFN-stimulated genes (ISGs) (such as IFI6, ISG15, SAT1, PMAIP1, p21/CDKN1A, and CCL2) [73]. It has been reported that members of the ISG protein family prevent infection before viruses can traverse the lipid bilayer of cultured cell, such as has been proven for influenza A virus and SARS coronavirus [74]. On the other hand, MSC display both direct and indirect anti-microbial mechanisms of action, which are complementary. Thus, they might act directly through the secretion of antimicrobial peptides, which are evolutionarily conserved small effector molecules (10–150 amino acids) found in organisms ranging from prokaryotes to humans [75]. These peptides include cathelicidin, defensins, cystatin C, elafin, and lipocalin 2 [76]. They mediate antimicrobial cell killing, which occurs by disrupting membrane integrity; inhibiting protein, DNA, or RNA synthesis; and interacting with certain intracellular targets [77]. In addition, these antimicrobial peptides can be active against certain pathogens that are resistant to conventional antibiotics, such as multidrug-resistant bacteria [78]. Specifically, cathelicidin is one of the factors produced by systemic MSC that significantly contributes to *Staphylococcus* killing [79]. However, the main mechanism of action is reported to be indirect via LL-37, a cleavage product of the cathelicidin, hCAP-18, originally found in the peroxidase-negative granules of neutrophils [80]. LL-37 has a broad range of antibacterial activity against both Gram-negative and Gram-positive bacteria [81,82,83]. In addition, LL-37 has antifungal [84] and antiviral activities [85]. LL-37 is also functionally linked to the modulation of Toll-like receptors (TLRs), which trigger the immunomodulatory activity of MSC [86]. Activation of TLR receptor in MSC is also by pathogen-associated molecules such as LPS or double-stranded RNA from viruses. TLR4-primed MSC after microbial molecule recognition secrete chemokines such as MIP-1α and MIP-1β, RANTES, CXCL9, CXCL10, and CXCL11, as well as IL-6, IL-8, MIF, and granulocyte-stimulating factor (GM-CSF), which promote the recruitment of neutrophils and monocytes [87,88,89]. Other soluble proteins from MSC that have a defensive effect against microbial are interleukin-10 (IL-10), prostaglandin E2 (PGE2), tumor necrosis and factor-alpha (TNF-α) [90], IDO [91], and interleukin-17 [92].

Recent *in vitro* and *in vivo* studies also reported that secreted products found in MSC-conditioned medium have a wide range of anti-microbial activity, including against *E. coli* and *S. aureus* [76,93], *E. epidemidis* [93], *Vibrio cholerae* [17,94], *P*. *aeruginosa* [95], *Mycobacterium tuberculosis* [96], *Acinetobacter baumannii* [97], and several *Candida* species [98,99], including effects against biofilm formation [100,101].

#### 2.2.8. Homing Effect

Stem cell homing refers to the characteristic that these ones can spontaneously migrate to the injured region when the body is wounded [102]. Although MSC reside in their biological niche of origin in physiological conditions, they seem to have the capacity to be mobilized in response to signals produced by injured tissues [103]. In fact, MSC have been proven to have the ability to home to traumatized areas after transplantation in *in vivo* studies. *In vitro* studies demonstrated that expression of chemotactic signals from hurting tissues, such as TNF-α [104], PDGFA [105], IGF-1 [106], HGF, and EGF [107], as well as the expression of chemokines for which MSC have receptors, such as adhesion molecules and, especially, several receptors, such as CC1, 4, 7, 9, and 10; CXC4 and 6; and CXCL12 [107,108,109], which stimulate the MSC attraction. With regard to this latter aspect, it is of note to say that MSC show variably to express multiple other chemokine receptors, which determine to which tissues MSC will migrate [102]. It is also key that MSC secrete MMPs, in particular MMP1, which has a role in tissue invasion in order to permit that MSC can traverse the endothelial basement membrane [110].

Further studies to enhance MSC homing effect could offer the advantage to reduce the number of required MSC for achieve therapeutic effects. In this sense, different methods have been proposed, such as genetic modifications, direct administration of MSC into the target tissue, cell surface modifications, *in vitro* priming, or pre-treatment of MSC [102]. On the other hand, MSC, by their inherent homing/targeting capacity, also offer the possibility to use these stem cells as carriers for certain drugs. For example, this strategy could resolve the non-selective cytotoxicity of chemotherapeutic agents. In this sense, a “trojan horse” biomimetic delivery strategy has recently been reported on, which uses MSC for photodynamic therapy and photothermal therapy against lung melanoma metastasis [111].

### 2.3. First Clinical Applications of the MSC

In addition to classic investigative applications of MSC, such as imperfect osteogenesis [112], graft-versus-host disease (GvHD) [113], Crohn’s disease, stroke [114], osteoarthritis (OA) [115], multiple sclerosis (MS) [116], liver fibrosis [117], or cardiovascular disease [118], the wide range of possible applications of MSC and/or their secretome is constantly expanding. These, on the basis of the positive results of preclinical studies, include the use of MSC in around of 1000 clinical trials over 10,000 patients (see ClinicalTrials.gov) in multiple indications as diverse as musculoskeletal defects; disorders of the immune system including auto-immune diseases, bone, heart, liver, lung, and kidney; or neurodegenerative disorders [1,119]. Besides this, it has been reported that MSC therapy does not have any unfavorable side effects on the patient. Safety of MSC therapy has been reported in a systematic review of several clinical trials involving over 1000 participants with intravascular MSC transplantation for different diseases, with follow-up of about 2 weeks to 30 months [120].

On the other hand, this increases in the clinical implementations of MSC being parallel to the growing evidence indicating a MSC dysfunction or depletion in systemic diseases, such as systemic lupus erythematosus (SLE), diabetes mellitus (DM), rheumatoid arthritis (RA), MS, idiopathic pulmonary fibrosis, Parkinson disease (PD), amyotrophic lateral sclerosis (ALS), psoriasis, myelodysplastic syndromes, and aging [2]. These data seem to indicate the importance of MSC in the tissular homeostasis.

Thus far, MSC therapy has been approved in different countries. Potential clinical applications of adult human mesenchymal stem cell therapy (Prochymal) were approved in Canada to treat acute GvHD in children. In Japan, the use of MSC was approved after the Act on the Safety of Regenerative Medicine and the Pharmaceuticals, Medical Devices and Other Therapeutic Products Act were introduced [121]. In 2018, the European Medicines Agency (EMA) recommended the approved of Alofisel to treat Crohn’s disease [122]. Overall, it appears that the use of MSC for cell therapy is becoming a reality. Nonetheless, MSC therapy in the United States has been approved by the Food and Drug Administration (FDA) in only very rare instances.

### 2.4. Limitations in the Era of Cellular Therapy with MSC

There are some discordant results in clinical trials based on MSC therapy for GvHD. However, historical clinical trials show positive results concluding that MSC are an effective therapy for steroid-refractory GvHD [113,123,124]. For example, a recent review of completed randomized clinical trials that used MSC for the treatment of GvHD found that MSC might have little or no effect [125]. As another example, early studies suggested improvement in cardiac function in the treatment of ischemic heart failure by using bone-marrow derived MSC. However, in subsequent clinical trials, there were no significant differences between MSC treatment and placebo [126].

The discrepancies found in the effectiveness of using MSC in clinical studies may be due to the overall quality of the study design [125], origin of MSC, tissue processing, donor gender, age, medical history, differences associated with manufacturing MSC in culture conditions, reductions in cell quality during *in vitro* expansion, administration routes, doses and dosing intervals, poor cell survival after *in vivo* transplantation, or inefficient homing capacity to targeted sites. All this limits the effectiveness of MSC therapy. In addition, an excessive inflammatory immune response, oxidative stress, and hypoxic microenvironments at sites of injury are also factors that restrict MSC survival and engraftment [20,127].

It is relevant to consider that although the MSC isolated from different tissues are alike, they differ functionally (proliferation capacity, transdifferentiation, immunophenotype or by both paracrine or microvesicle mechanisms via secretome-derived products) depending on the origin of the tissue [63,128,129], and which are maintained in culture conditions [130]. In fact, proteomic comparison of MSC-derived secretome from different tissue sources has revealed differing profiles and capabilities. For example, MSC-derived secretome from adipose tissue, bone marrow, dental pulp, and Wharton’s jelly present different protein compositions [131,132]. It has been also reported that WJ-MSC secrete greater amounts of proinflammatory proteins and growth factors, while those derived from adipose tissue have an enhanced angiogenic profile and secrete greater amounts of extracellular matrix proteins and metalloproteases [133]. Donor age is an important factor affecting MSC efficacy. MSC grown from neonatal tissues show a longer lifespan and higher proliferation rate and differentiation potential when compared to adult tissues [134]. Furthermore, MSC derived from diseased donors may show negative clinical outcomes when used for therapies [135]. With regard to manufacturing, MSC are cultured for long periods of time to obtain clinically relevant cell numbers, which results in important changes in gene expression and clonal selection, thus affecting biologic properties, including those involved in tissue regeneration. In addition, cell subculture requires the use of proteolytic enzymes, which may damage the cells [136]. Other important aspects to take into account are the components of culture media that may affect cell phenotype, such as the damage caused by cryopreservation and subsequent thawing, as well as oxygen concentration. High oxygen levels may compromise the therapeutic benefits of MSC. Native MSC tissue environments range between 1 and 7% O_2_. During culture, cells sense an oxygen concentration of 20%, which may cause oxidative stress affecting viability, and eventually senescence [137,138,139].

On the other hand, there are several issues related to cell therapy, such as several safety considerations potentially associated with the transplantation of living and proliferative cell populations, including immune compatibility, tumorigenicity, emboli formation, and the transmission of infections [35].

No less important drawback is the economic cost of these therapies. It has been estimated that a mere stem cell therapy can range from USD 4000–8000 in the USA and the cost for culturally expanded cells ranges from USD 15,000–30,000 [140]. Although compared to monoclonal antibody therapy, the costs of MSC secretome probably appear to be lower (Tocilizumab costs USD 355.000 for a single dose) [141], it needs further research to evaluate the cost-effectiveness of cell-based therapy and to guarantee sustainable access for patients and the general population [142].

## 3. Non-Cultured Cell Strategy Alternative

A variation of the uncultured cell strategy relies on the administration of microfragmented adipose tissue, in which the genuine microenvironment of presumptive MSC is maintained intact [143]. With the tissue undisturbed by enzymatic digestion, cells sustain higher secretory activity, releasing abundant cytokines and growth factors [144].

In general, transplantation of uncultured cells may be ideal to improve clinical outcome, although numbers of cells obtained are lower than in culture conditions and may not be enough for proper treatment in some indications.

Finally, since ubiquitous presumptive MSC have been identified in perivascular spaces that become recruited and reprogrammed in adverse disease/injury conditions, an ideal alternative to MSC administration might be the targeted pharmacologic mobilization of these cells in situ.

## 4. Beginning of the Era of Therapy Based on Secretome of MSC

The initial concept of MSC therapy was based on the fact that they have the ability to homing to injury sites and differentiates into different cell types contributing to tissue regeneration. However, several studies have revealed that the implantation time of MSC is usually too short to have an effective impact [145,146]. Indeed, it has been reported that <1% MSC survive for more than one week after systemic administration [147,148,149], and their contribution to new tissue formation is generally minimal [150]. Although several studies indicate that MSC exercise many biological effects by promoting cell-to-cell interactions and cellular proliferation [151,152], the accumulated experience indicates that the beneficial effects of MSC are mainly via the secretion of paracrine factors. These soluble factors include proteins (growth factors and cytokines) and EVs. Due to the regenerative, anti-inflammatory, and anti-oxidative stress and angiogenic and anti-apoptotic power from these biological products, MSC secretome may be considered a good candidate for a new medical biotechnology [153]. This strategy avoids the problems derived from using the stem cells themselves, among others, for example: (i) the safety problems derived from the transplantation of proliferating living cells are solved, including immunological incompatibility, tumorogenicity, the formation of emboli, transmissible infections, and the potential entry of MSC into senescence; (ii) unlike cell therapies, secretome can be better evaluated in terms of safety, dose, and potency, in a similar way to conventional therapeutic agents; (iii) secretome can be stored without the need for the application of potentially toxic cryopreservative agents; (iv) the use of products derived from the secretome, such as the conditioned medium or exosomes, is cheaper and more practical for clinical use, since the use of the secretome could avoid the time and costs associated with expansion and maintenance of clonal cell lines. This is owing to the fact that secretome for therapies could be prepared in advance in large quantities and be available for treatments when necessary [35,154].

Preliminary studies suggest safety and efficacy of MSC secretome, where dosing may include topical, intravenous, and oral application [155]. Products of the secretome could take over from the attempt of therapies based on the administration of growth factors. Numerous studies have found that growth factor therapies (EGF, PDGF, KGF, GM-CSF, etc.) have positive results in many animal models of diseases, such as wound repair. However, its translation into clinical products has been limited due to the amount necessary of growth factors, the expense of manufacture, and the lack of clinically relevant benefits [156,157,158,159]. Nevertheless, the secretome as a whole may have the limitation of representing too biologically complex a product, which can make it difficult to identify a robust mechanistic explanation for its therapeutic effects. Thus, a possible alternative may be based on the alternative of using a more specific part of its components, such as EVs.

### 4.1. Extracellular Vesicles from MSC: Tropism, “Trojan Horses”, and “Fire Cars”

EVs of the MSC secretome are generating an extraordinary interest as an encouraging alternative to exploit MSC properties. EVs can be classified as (i) exosomes (30–120 nm in diameter), which originate within the cell in endosomal compartments called multivesicular bodies; (ii) microparticles (150–1000 nm in diameter), caused by blistering outward from the plasma membrane and subsequent release after proteolytic cleavage of the cytoskeleton; and (iii) apoptotic bodies (500–2000 nm in diameter), which are released during the programmed cell death process. Among these EVs, exosomes stick out due to their functional importance, whose biogenesis process consists of four phases: initiation, endocytosis, multivesicular bodies, and release [160,161]. EVs are membrane-bound phospholipid particles secreted by cells, containing a wide range of different components, such as RNA, lipids, proteins, cytokines, chemokines, interleukins, integrins (CD81, CD63, and CD9), transport proteins (annexins and Rab GTPases), signal transduction factors (kinases), cytoskeletal proteins, and metabolic enzymes [162]. Through the horizontal transfer of all these biologically active factors, exosomes represent an intercellular communication pathway that constitutes a principal part in mammalian cell connection [161,163]. These mechanisms include binding to surface receptors to activate signal cascades, internalization of surface-bound exosomes, and fusion with the cell to deliver material directly to the cytoplasmic membrane and cytosol [164].

It is known that EVs carry intact mRNA that can be transferred horizontally and translated in the recipient cells [165]. However, several studies also found non-coding RNA (ncRNA) and components RNA, including both small non-coding (<200 nucleotides) and long non-coding (≥200 nucleotides) RNA, which operate essential regulatory function in a number of physiological processes on recipient cells. MicroRNA (miRNA) represents the most widely studied type of small ncRNA. It has been reported that between 30 and 80% of protein-coding genes are regulated by miRNA. This is primarily achieved through the complementary binding of miRNA to a target mRNA sequence, resulting in either mRNA degradation or translational repression [166]. Circular RNA (circRNA) are closed-loop structures representing a unique class, highly stable form of non-coding RNA present in EVs [167]. There is increasing evidence pointing out that circRNA act as post-transcriptional regulators in several biological processes including cellular repair [168] and cancer progression [169].

Experimental studies demonstrate the therapeutic benefit of these products in a wide range of conditions or diseases [5]. In addition, exosomes show important advantages for their application in therapies: they are smaller, less complex and less immunogenic than their progenitor cells, since they have a lower content of proteins bound to the membrane [170]. The production and storage of exosomes are easier than for their parent cells. Furthermore, other advantages of exosomes include a longer half-life in the bloodstream [171], ability to cross the blood–brain barrier, and tropism towards inflamed tissues and tumors [172,173].

On the other hand, it is possible to improve the release and capacity of exosomes through various strategies, such as prolonged cultivation and maintaining cells at low pH [174] or a low O_2_ tension [175,176,177,178]. Preclinical studies have demonstrated the safety of exosomes derived from MSC, given the possibility of their massive scalable production at clinically relevant levels [151]. Currently, around 210 trials based on the therapeutic application of exosomes have been launched, of which four are active and cover conditions as varied as COVID-19 (Coronavirus Disease 2019), lymphoma, and breast cancer. They are all in phase I or phase II, and no results have come out thus far. This information is available from www.clinicaltrials.gov (accessed on 29 March 2021).

Interestingly, there is experimental evidence indicating that exosomes can be manipulated with certain ligands or proteins on their surface to improve their targeting capability, as well as loaded with therapeutic products. In this way, they can be induced to behave like “trojan horses” and “chariots of fire”. Thus, for example, exosomes encapsulated with miR-379 have improved migratory capability in vivo to relocate to the tumor site of breast cancer displaying antitumor effects. In addition, methotrexate-loaded EVs functioning with a synthetic multifunctional peptide have been shown to facilitate the membrane receptor-mediated internalization procedure in glioma experimental models [179]. On the other hand, it has been reported that human MSC treated with sub-lethal concentrations of paclitaxel have an antitumor effect against several human cancer cells, such as A549 lung cancer, SK-OV-3 ovarian cancer, and MDA-hyb1 breast cancer cells [180].

### 4.2. Potential Side Effects and Limitations of Therapies Based on Secretome from MSC

There is limited information on the potential risks of therapy based on secretome products. These potential risks seem related to the administration of exogenous biological products, but these appear reduced compared with cell-based therapies.

Apparently, safety concerns could be related to the immunogenicity and immunosuppressive properties of the MSC secretome. Although the secretome is known to contain extracellular vesicles that can be immunogenic, this potential immunogenicity has been found to be less than that of its parent MSC [181]. On the other hand, considering that the MSC secretome has immunosuppressive properties, and therefore having been described as one of the main mechanisms of action of when managing autoimmune diseases [182], its use might theoretically increase the risk of infection, immunodeficiency, and tumor growth in treating patients [183]. As discussed above, there are data suggesting that the immunoregulatory effects of MSC in vitro depend on the status of the inflammatory microenvironment. Although this condition could also exist in in vivo conditions, it will be necessary to define, depending on the clinical circumstances and the therapeutic interest, the optimal amount of secretome to achieve the appropriate balance between safety and efficacy.

With regard to constraints in production, there are two technical aspects, secretome resources and instability of secretome, which require further solutions. For example, it is estimated that the number of MSC required to produce sufficient quantities of secretome for an equivalent effect on acute wounds is about 10–25 times higher than directly administered live cells [184]. This highly elevated demand in production enhances manufacturing, quality control, derivation, and validation costs because the function of these cells may change with repeated passages. In addition, potential issues in secretome therapy may be the possible instability and lack of potency of the secretome.

However, there are methods to address all these limitations, which are outlined below, and include more optimal and productive methods for expanding cell cultures and increasing the functional potential of MSC and their derivative products from their secretome.

## 5. New Horizons for Clinical Applications of MSC

Despite of the weaknesses of MSC-based therapies, both the classical research uses of MSC and the wide range of possible utilities of MSC and/or their secretome are on the rise. These, on the basis of the positive results of preclinical studies, include some examples of frequent and varied diseases as the following.

### 5.1. MSC Applications as Regenerative Therapy

#### 5.1.1. Skin Ulcers

The skin is the largest organ, accounting for 16% of the body weight, protecting the organisms from external physical (e.g., ultraviolet radiation), organic, or biological aggressions [185]. There are many diseases and processes such as atopic dermatitis [186]; psoriasis [187]; or, especially, chronic skin wounds [188], which need new therapeutic approaches, constituting MSC as a possibility.

In addition, around 50% of DM-related foot ulcers are refractory to current therapies [189].

Autologous BM-MSC have been administered to chronic cutaneous ulcerations [190], not only diabetic foot ulcers [191] but also pressure ulcers [192] or radiation burns [193], resulting in accelerated wound closure and improved healing properties. All of these findings from preclinical and clinical studies demonstrate that MSC could be a promising resource for the highly demanded skin regeneration [194].

#### 5.1.2. Bone Regeneration

Osteoporosis is an extremely common orthopedic condition. For example, it is estimated that approximately 12.3 million individuals in the United States are expected to be affected by this condition, which favors the development of secondary fractures [195]. In addition, these fragility fractures have the risk of failure of bone regeneration and consequently non-union, deformities, and chronic pain, which often require an invasive surgery with associated risks [196,197] and a huge economic spending [198].

BM-MSC have shown a high osteogenic differentiation capability and are the most common types of MSC that have been used for osteoporosis [199,200]. It was also recently reported that BM-MSC-derived exosomal miRNA-150-3p promotes osteoblast proliferation and differentiation in osteoporosis [201]. For all of this, stem cell-based therapy and their secretome-derived products are considered as a new approach to regenerate the bone tissue [202].

Dental pulpal disease is also one of the most prevalent illnesses that millions of people are suffering from all around the world [203]. The current therapy is to remove the pulpal tissue and replace it with synthetic materials such as resin and gutta-percha. However, these materials are not capable of replacing biological functions of lost tissue, leading to the reduced mechanical properties and reduced vitality of the teeth. Therefore, stem cell-based therapies would be an improving approach to repair or replace the damaged or lost tissues of teeth in order to recover the morphological and biological functions [204]. Recently, it was shown that UC-MSC possess the ability to differentiate into odontoblast-like cells under the microenvironment induced by a liquid extract of human treated dentin matrix in vitro [205]. On the other hand, we consider the possibility that MSC secretome-derived products, such as conditioned medium and/or exosomes, may be a new strategy to induce dental stem cells to dent pulp regeneration, which will require further well-designed investigation.

#### 5.1.3. Osteoarthritis

At present, conventional OA therapeutics are often inadequate to alleviate the symptoms of the disease. Therefore, it is necessary to formulate new therapies that reduce inflammation and promote cartilage regeneration. Interestingly, it has been reported that MSC exhaustion and functional decline may be implicated in the pathogenesis of OA [206].

This is a growing area of research, and several studies have reported on the clinical efficacy of MSC in OA. In recent years, an increasing number of studies demonstrated the beneficial effects from EVs, particularly exosomes from BM-MSC, in in vivo studies of OA.

#### 5.1.4. Heart Repair

Cardiovascular diseases are the leading cause of mortality and morbidity globally, representing approximately one-third of all deaths every year. Despite recent pharmacological and mechanical advances having significantly contributed to the sharp decline in death rates [207], myocardial infarction (MI) continues to be a major cause of mortality and morbidity worldwide. In a global report on the incidence of disease and injury, it was estimated that around 10.6 million cases of MI caused by ischemic heart disease had occurred in 2019 alone [208]. It is calculated that after myocardial infarction, up to 1 billion cardiac cells die in response to ischemic injury, leading to reduced cardiac function, scar formation, and heart failure [209]. This is due to the fact that, compared to other organs, the heart has a low regenerative capacity [210]. Cardiac transplantation remains the only true cure for failing hearts [211], but the limited number of available donors limits therapeutic solutions. Thus, the possibility of myocardial regeneration by using cell-based therapy represents a promising field of research [212].

Despite the highly promising results in animal models, there is modest benefit observed in human clinical trials of acute MI [213,214] or chronic ischemic cardiomyopathy [215]. There is some aspect to explicate these conflicting results. The lack of benefits has been attributed to the differences in type, dose of injection, route of administration (for example, via intracoronary, epimyocardial, intravenous, intraperitoneal, or intramuscular injection), or administration time of MSC [216].

Experimental studies demonstrated that MSC-conditioned medium enhances cardiac progenitor cell survival after hypoxia-induced injury [217,218,219]. In addition, although their exact components of the cargo that provide cardioprotection are yet to be discovered and characterized, several experimental studies have outlined that exosomes from MSC reduce the infarct size and improve post-myocardial infarct cardiac function [220].

### 5.2. MSC Applications as Anti-Inflammatory Therapy

#### 5.2.1. Neurodegenerative Disorders

The traditional treatment of neurodegenerative diseases has not yet achieved ideal results, and early diagnosis is hindered due to the lack of effective biomarkers [221]. Consequently, these diseases, such as Alzheimer’s disease (AD), PD, MS, brain cancer, ischemia, traumatic brain and spinal cord injury, and viral infections of the CNS represent the leading causes of death and disability worldwide. In addition, as the incidence of neurodegenerative diseases rises in aging populations, this burden is expected to substantially increase due to the associated augment in life expectancy [222,223,224,225].

Among the different studies under investigation, those in MS are distinguished, where the administration of human UC-MSC or human BM-MSC have revealed an immunomodulatory effect able to provide clinical stabilization, an improvement of the symptoms, and a reduction of the onset of relapse [226,227,228,229]. With regard to amyotrophic lateral sclerosis, several studies have reported that the injection of autologous human BM-MSC into the spinal cord causes an upgrade of functional assessment scale scores and a slowing of disease progression [230,231,232].

Current studies have also proved that the administration of MSC promotes recovery from traumatic brain injury due to oxidative stress reduction [233,234], reduced mortality rates [235], and the size of the infarct area [236] after ischaemic stroke. In addition, MSC-based therapies represent an exciting neuroprotective and neuroregenerative strategy for spinal cord injuries [237], which are associated with tremendous physical, social, and financial costs for millions of individuals worldwide.

It is also of note to mention prevailing studies pointing to a potential therapeutic effect of secretome from MSC in brain diseases. Thus, for example, it has been recently published that the secretome derived from MSC pre-conditioned in vitro in an AD environment (MSC-CS), also intranasally, in APP/PS1 mice, completely re-established mouse memory and remarkably changed neuropathology at multiple crucial levels in very advanced AD stages [238].

##### Ophthalmologic Diseases

Summing up, results obtained in a large number of experimental studies revealed that beneficial effects of MSC and their secretome in glaucoma therapy relied on their capacity for neuroprotection and RGCs regeneration [239].

In recent years, there have been various studies in the literature on the therapeutic effects of MSC on the damage in retinal cells [240,241].

#### 5.2.2. Lung Diseases

It has been reported that 3.2 million people died from chronic obstructive pulmonary disease in 2017 [242], and it is estimated that this disease will be the third most important cause of death worldwide by 2030 [243].

Morphological and functional alterations of the lung MSC have been described associated with processes related to aging, acute lung injury, chronic obstructive pulmonary disease, or bronchopulmonary dysplasia [244,245,246]. In addition, it is known that MSC secrete plenty of molecules with paracrine effects that promote regeneration of pulmonary alveoli (angiopoietin 1 (ANGPT1), HGF, EGF, KGF, and VEGF [247]), regulate immune cells toward an anti-inflammatory phenotype (TGF-β, IL-1RA, IL-10, NO and IDO [248,249]), prevent epithelial–mesenchymal transition of alveolar epithelial cells in the context of lung injury [250], and improve bacterial clearance stimulating phagocytosis activity of macrophages through the secretion of antimicrobial factors such as peptide LL-37 and lipocalin-2 [87]. Accordingly, experimental lung disease models, such as chronic obstructive pulmonary disease, asthma, bronchopulmonary dysplasia, idiopathic pulmonary fibrosis, and acute lung injury, illustrate the therapeutic efficacy of MSC [251] or their exosomes [252]. On the other hand, clinical phase I and II studies based on MSC administration also demonstrate preliminary safety results in patients suffering these processes [253,254,255,256,257]. Recently, urgent clinical trials demonstrated the certainty and therapeutic efficacy of MSC in COVID-19 patients. This beneficial effect of MSC was attributed to their anti-inflammatory mechanism against the cytokine storm associated to COVID-19 [258]. In this day and age, new clinical trials are on by using MSC from several origins and with different administration routes [259], and given the extremely serious and urgent situation of the coronavirus pandemic (SARS-CoV-2), MSC as an alternative for the treatment of critically ill patients is considered under compassionate use protocols.

#### 5.2.3. Infectious Diseases

Another important problematic perspective in medicine is the treatment from infections. This is mainly due to the excessive exposure of bacteria to antibiotics, which has altered bacterial genomes and has led to the development of multidrug resistance in bacteria [260], the reduced susceptibility to common antifungal drugs used for treating these diseases [261], or the emergence of new and dramatic virus pandemics such as SARS-CoV-2 [259,262]. MSC and/or their derived product may be one possible alternative. In addition, recent studies highlight the interest in improving antimicrobial effect by combining of MSC secretome-derived products with nanoparticles. Thus, for example, research has shown the synergic effect of MSC-conditioned media coupled with chitosan nanoparticles against multidrug-resistant *Vibrio cholerae* [94]. On the other hand, MSC can produce a direct antiviral effect by secreting antibacterial peptides and proteins (IDO, IL-17, etc.) and activate a large number of antivirus genes that can encode protein structures that prevent viruses from invading cells [263]. Additionally, MSC can also exert an indirect antiviral effect by regulating the dynamic coordination of pro-inflammatory and anti-inflammatory elements of the patient’s immune system and promoting the activity of phagocytes [78].

Interestingly, MSC are usually resistant to viral infection due to their expression of ISGs such as IFITM, IFI6, ISG15, SAT1, PMAIP1, p21/CDKN1A, and CCL2 that preempt viral infection [73].

### 5.3. Cancer

The worldwide number of cancer patients is expected to increase from 14 million in 2012 to more than 19 million in 2025 [264]. In addition, cancer will be an important cause of morbidity and mortality, including the adverse effects derived from their treatments (chemotherapy, radiotherapy, hormonal therapy, and immunotherapy). Therefore, new therapeutic alternatives are necessary today.

There are specific types of MSC that show genuine antitumor properties such as those from uterine origin (endometrial tissue, uterine cervical tissues) as well as other reproductive tissues (amniotic fluid, placental chorionic villi, and umbilical cord). In the same context, in the human uterine cervix, which is permanently in contact with a mildly aggressive environment, including bacteria and oncogenic variants of the papillomavirus family, a process known as “squamous metaplasia” takes place. Our hypothesis is that human uterine cervical stem cells (hUCESC), embedded in the uterine cervical stroma, have a unique ability to hold all these extreme biological hazards under control through the regulation of proliferation and oncogenic transformation. In support of this hypothesis [265], a potent anti-tumor effect of this MSC type has been shown [67].

On the other hand, the tropism for tumors that characterizes MSC makes them potential candidates to be applied in future clinical trials as selective vehicles for drug delivery. This homing condition from MSC toward tumors, together the fact of that MSC are relatively resistant to cytostatic chemotherapeutic agents, motivated the use of drug-loaded MSC to target cancer [266,267,268,269]. Moreover, research has investigated the strategy of producing MSC genetically manipulated to express specific enzymes, such as cytosine deaminase or herpes simplex virus thymidine kinase, which converts inactive systemically administrated prodrugs, such as fluorouracil and ganciclovir, into active cytotoxic agents, which increase tumor-directed chemotherapy activity minimizing systemic toxicity [270,271]. Other possibilities are to use MSC as carriers of oncolytic viruses that destroy cancer cells [272,273], or to carry out genetic modifications of MSC to express anticancer bioactive molecules [274].

On the other hand, the use of secretome-derived products from MSC, such as exosomes, is also a further possibility. Exosomes maintain tumor-homing ability of their parental cells [275] and have longer circulating half-time [171], while cancer cells internalize higher percentage of exosomes compared to normal cells, better crossing through the blood–brain barriers [173], and they can be easily manipulated and modified with certain ligands or proteins on their surface in order to improve their targeting capability [276]. In addition, exosomes have been loaded with cytotoxic chemotherapy agents (paclitaxel, doxorubicin, or gemcitabine) [269] or miRNA (miRNA-133b [277], miRNA-148-3p, miRNA-205 [278], or miRNA-1231 [279]), which inhibit the activity and progression of several human carcinomas.

## 6. Need of New Strategies of MSC Production

On the basis of all the above information on the potential new demands for MSC-based therapies and the existing limitations on their in vitro production, we cope with the need to achieve unprecedented tactics that increase and improve their in vitro expansion and the production of derivatives of their secretome.

**Figure 2:**
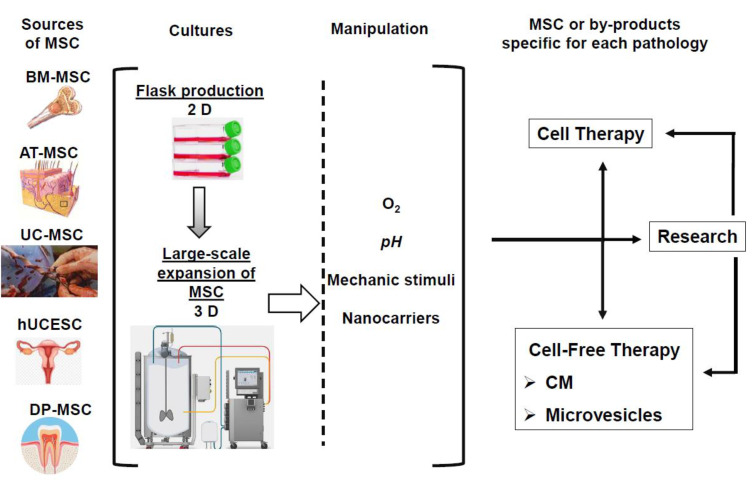
Important concepts for mesenchymal stem cells (MSC) production strategies. It is necessary to consider, for each application, tissue origin of MSC, system employed for in vitro expansion, and the final product for the therapy.

These actions have to include the optimal selection of the ideal MSC, in terms of the donor and tissue origin for each application, the establishment of adequate in vitro expansion standards for the MSC, as well as the implementation of functional potency tests of the obtained products (Figure 2).

### 6.1. The Ideal Cell for Every Application

Considering the functional heterogeneity of MSC and the factors that influence it, not only related to the donor, but also to their origin in different biological niches, we understand that selecting the most appropriate MSC for each indication will be necessary in the future.

It is known that patients affected by systemic diseases such as DM, obesity, SLE, and RA can have functional alterations in their MSC, which must be taken into account so as not to consider them as ideal donors. In addition, the aging of MSC during expansion is a key limiting factor due older cells losing competence to behave as stem cells and having a tendency to enter senescence or even to undergo transformation. Aging MSC are more likely to activate a senescence-associated secretory phenotype and produce pro-inflammatory cytokines such as IL-1, IL-6, and IL-8, which inhibit the regenerative process [280]. It is also known that culture-expanded MSC, in general, lose their self-renewal capacity and multipotency progressively [281,282], which is a major limitation for research and potential treatments [1,283]. It has also been shown that MSC are not able to carry out more than 30–40 population doublings, while immortalized MSC are able to reach more than 200 population doublings [284,285]. Immortalization, which requires repression of p53- and Rb-mediated pathways and telomere maintenance, allows these cells to increase their proliferation rate and to avoid senescence, while maintaining mesenchymal phenotype and multipotency. MSC immortalization can be achieved by the combination of transduction of immortalizing genes such as simian virus 40 large T antigen (SV40LT), which promote cell cycle progression and human telomerase reverse transcriptase (hTERT) and prevent telomeres shortening [286]. In addition, both morphology and functionality of MSC were found to not change after transduction of immortalization genes [285,287].

On the other hand, it is known that functionally from MSC differs with regard their tissular origin. Thus, for example, differences were reported between AD-MSC and BM-MSC in terms of proliferation, differentiation, or paracrine mechanisms, such as the secretion of pro-angiogenic molecules and extracellular components of MMPs [63]. In addition, there are new known supplier of MSC that may be attractive for specific indications, such as MSC from reproductive tissues by their anti-tumor activity [67,265], hUCESC for their antifungal activity [98], or MSC from dental pulp by neurological disorders [288].

Genetic manipulation of MSC using application of replication-defective viral vectors, such as lenti- and adenoviruses, provide a possibility of improving some of their capabilities. Thus, there are data arguing that the incorporation of anti-inflammatory genes to MSC (for example, IL-10, HGF, IDO, or Foxp3) could improve their therapeutic potential. Similarly, it has been reported that induced overexpression of several factors leads to better apoptotic tolerance and cell survival, as well as more angiogenic, neuroprotective, osteogenesis, or anti-cancer activities [63]. Nevertheless, despite all these positive data on genetic manipulation of MSC, there are several barriers to their clinical application. The introduction of replication-defective viral vectors, such as lenti- and adenoviruses, is associated with toxicity, immunogenicity, and potential tumorigenicity [289]. More recently, several studies have demonstrated that the clustered regularly interspaced short palindromic repeats (CRISPR)-Cas system may improve the therapeutic potential of MSC [290]. Thus, for example, research has reported the use of engineered BM-MSC overexpressing IL-10 using CRISPR activation to treat myocardial infarction in an experimental model [291].

### 6.2. MSC In Vitro Production

Although MSC are widespread in practically all organs and tissues, they are found in minute quantities. Thus, as an example, MSC isolated from bone marrow only occupy approximately 0.001–0.01% of mononuclear cells for healthy adults [292], 0.3% from umbilical cords [293], or 1.2% from human adipose tissue [294] for healthy adults. These amounts of MSC are very far from those required in clinical applications, which are around 2 × 10^6^ cells/kg of body weight per dose [295,296]. Furthermore, for certain patients and diseases, multiple administrations of up to several hundred million MSC are needed to achieve the effective therapeutic outcome [296]. Therefore, the in vitro expansion of MSC is necessary about many weeks before to achieve sufficient cells for cell-based therapies.

#### 6.2.1. Flask Production

Most of the centers use the classic production system in T-flasks. Nonetheless, this type of method is only suitable for treating a small number of patients. For example, it was estimated that a production of 30 T-flasks each with a growth surface of 175 cm² would be required per patient, assuming each patient is dosed with 416 million cells and the harvesting efficiency is 8 × 10^4^ cells/cm² [297]. However, for larger clinical trials with >100 patients, the resources required for cell culture would become insupportable (assuming the conditions stated above, a trial with 140 patients would require 4200 T-flasks filling 32 standard 160-L incubators and 9 full-time personnel to handle the cells) [280]. In addition, previous studies also reported that MSC proliferation and differentiation potential decreased when they reached a higher passage number [298]. Thus, identification of an effective large-scale expansion technique is critical to obtain the huge number of cells in a short period of time and in a cost-effective manner without compromising the cell quality. All of them are requirements for the demand of thousands of in vitro and in vivo studies, late-phase clinical trials, and future commercialization, which is increasing exponentially [297].

#### 6.2.2. Large-Scale Expansion of MSC

There are several bioprocessing strategies for large-scale production, such as multilayered flask, spinner flask, roller bottle, or bioreactor, which are used for expansion of MSC from different sources (AT-MSC, UC-MSC, WJ-MSC, BM-MSC, periosteum-derived MSC (PD- MSC), VC-MSC), dental pulp-derived MSC (DP-MSC), and fetal MSC (F-MSC) [296].

Considering that the production of adherent MSC depend on the surface area, it is key to achieve a maximum surface area in which the original biological characteristics of phenotype and potency of the MSC will be preserved. A multilayered flask is a specially designed culture flask that consists of multiple layers with a large surface for cell culture. It has been described that multi-layer vessels can produce MSC in an amount more than 100 times more than simple T-flasks [299]. For example, CellSTACK has a surface area ranging from 1272 cm^2^ for two-chamber to 3180 cm^2^ for five-chamber. Nevertheless, this system has drawbacks, such as the fact that real-time observation of cell morphological changes may not be applicable under a regular microscope, which is a static cell culture system. Spinner flask and roller bottle are dynamic culture systems that create shear stress to cells as it involves the mechanical agitation of the culture medium or culture vessel to allow for more efficient nutrient transfer. However, all of these systems are manual bioprocessing strategies with lower efficiency [300].

Alternatively, automated well-controlled bioreactors provide efficient mixing in a closed system for large-scale expansion in lot size at reduced labor and time [297,300]. The expansion of MSC in bioreactors permits us to advance in the quality of the product in several aspects, such as commercial manufacturing; greater traceability due to control and monitoring; possibility of elimination of errors and operator-related contamination; avoiding batch-to-batch variability; and, finally, abrupt fluctuations in pH, oxygen concentration, or nutrient gradients caused by manual medium exchange. Many types of bioreactors, including hollow fiber bioreactor, stirred tank bioreactor, vertical wheel bioreactor, and multiplate bioreactor have been tested for large-scale expansion of MSC. Stirred tank reactors are the most widely used devices for large-scale MSC expansion [301]. There are some key technological aspects to consider in these more advanced types of cell culture expansion systems, such as the use of microcarriers, hydrodynamic parameters, and agitation. Microcarriers provide a high surface-to-volume ratio for high-density cell culture with a cost of goods reduction. Microcarriers are small beads that increase the surface area available for cell attachment per unit volume. These structures, with size ranging from 100 to 300 μm in diameter, are made of diverse materials, such as polystyrene, dextran, cellulose, gelatin, glass, or decellularized tissue, with different surface properties [302,303]. Thus, these systems allow for the growth of cultures in 3D, which brings many advantages, especially inducing 3D spheroid of MSC in suspension. This microcarrier suspension culture includes advantages such as the scalable design, homogeneous culture environment, real-time monitoring of cells and medium, and the feasibility of maintaining a long-term culture via bead-to-bead transfer without enzymatic treatment/passaging [304,305]. Moreover, due to the high surface to volume ratio, less culture medium (a main cost driver) is used in MSC microcarrier bioprocessing.

Compared to those from planar culture, many groups have reported that a microcarrier culture of MSC improves their osteogenic and chondrogenic differentiation potential [306,307] and facilitates neuroregulatory function. Additionally, differentiation of neural progenitor cells [308] showed strong antioxidant effects and protected cell viability during oxidative stress, as well as enhancing their anti-inflammatory and immunomodulatory properties [309]. At the same time, stem cell spheroids have higher engraftment efficiency and survival at transplantation sites, exhibit elevated Bcl-2 levels (anti-apoptotic) and diminished Bax levels (pro-apoptotic) [310], and show higher levels of angiogenic growth factors (VEGF, FGF, angiogenin, and HGF [311]) compared to monolayer-cultured cells.

The parameters grouped under the term hydrodynamics refer to the potential impact of aeration and agitation. Aeration is required to supply oxygen to the MSC, which, in addition to affecting oxygen saturation, also generates forces that cause physical stress. In T-flasks, aeration is achieved by the diffusion of oxygen through the surface of the medium, whereas bioreactors must be actively aerated by, e.g., bubbling the gas into the liquid. The bursting gas bubbles generate strong forces that can damage cells [312]. Agitation in bioreactors is generally achieved with impellers, which help to disperse gas, but also maintain a homogenous suspension of cells and nutrients. The creation of a homogenous environment is advantageous because it avoids gradients of pH, nutrients, or waste products.

Large scale of biomanufacturing represents a great advantage over classical 2D cell culture systems. However, they can still represent handicaps, such as non-homogeneity of the culture systems, nutrition depletion, and residue accumulation due to high cell density and when the interstitial flow is insufficient [313]. Furthermore, we have to consider that microcarrier-expanded MSC show differences with planar culture-expanded cells in size, morphology, proliferation, viability, surface marker, gene expression, differentiation capacity, and secretion of cytokines. Thus, a better understanding of the bioprocessing parameters that influence MSC therapeutic efficacy is yet essential. With regard to this, there are many ex vivo possible MSC modifications to optimize bioreactor conditions to maximize MSC quantity without sacrificing quality and therapeutic potency [314].

#### 6.2.3. Ex Vivo MSC Modifications: Toward More Specific Therapeutic Applications

Ex vivo modifications to enhance the therapeutic interest of MSC in cultures include oxygen and pH, or pre-conditioning with inflammatory cytokines.

MSC are aerobic cells, and any culture vessel must therefore ensure an adequate supply of oxygen. However, the oxygen saturation in standard T-flasks (21% O_2_) is far removed from nature (5–7% O_2_) [315,316]. MSC therefore tend to be over saturated with oxygen, which can increase the concentration of damaging reactive oxygen species (ROS). Several studies have confirmed that hypoxia enhances MSC proliferation, stabilizes their cell fate, and prevents apoptosis by reducing the levels of caspase-3 [317]. However, rather than imposing hypoxia by preconditioning the cells, it may be better to impose hypoxia during the entire expansion phase, because this mimics their natural niche [318].

Several pieces of evidence show that MSC cultivated at low oxygen concentrations improves several therapeutic effects, such as expression of higher levels of pluripotent and proliferation markers [319,320], increase in the secretion of cytokines and growth factors in transplanted stem cells [321], improvement of angiogenesis [322], migration to the site of injury [323], and anticancer effects [177].

Typically, in vitro expansion is carried out at 37 °C and neutral pH (7.2–7.4). Although the expansion of MSC has been achieved in the pH range 7.5–8.3 [324], it is unclear how significant variations in pH influence MSC metabolism and whether this affects their secretome. The optimum temperature and pH must be evaluated for each MSC product.

Another pre-conditioning tactic to improve MSC therapeutic benefits includes exposure to an inflammatory environment in the presence of inflammatory cytokines, such as IFN-γ [325] and TNF-α [326]. Treating MSC with these inflammatory cytokines or their combination, their secretion of anti-inflammatory biomolecules increases, and their immunosuppressive function improves [63].

These data indicate the possibility of modulating the capacity of MSC and their secretome, according to different chemical or molecular stimuli. In such a way, we could conceive the possibility in the future of adapting the potentiality of the secretome of MSC to the optimal therapeutic applications that each specific pathology demands.

#### 6.2.4. Standardization and Functional Tests Research for Specific Applications

Recently, Stroncek et al. described functional and molecular differences between the MSC of three patients produced in five different laboratories, despite the fact that the origin of these cells was the same [327]. These situations occur because, today, there is no standardized protocol for each one of four key steps of the MSC manufacturing process: donor MSC selection and collection, maintenance and transport of the MSC from the collection site to the processing site, culture strategy (i.e., plating cells, passaging the adherent cells, and harvesting the MSC), and cryopreservation and storage of the manufactured MSC. In many cases, equivalent variables are used for different applications.

In the current situation of allogeneic therapies, MSC manufactured from one or several selective donors are used as a universal drug for multiple patients. However, many studies have reported about both donor-to-donor and tissue source variations [328,329,330]. In addition, MSC are isolated from a number of different tissue source materials and, as it was mentioned above, there is functional heterogeneity with regard to their origin. On the other hand, MSC are generated with different culture or preconditioning strategies [331]. Although only a few studies explored the impact of bioprocessing parameters on MSC therapeutic potency [308,332,333], it was reported that, for example, variable in vitro expansion strategies have a stronger impact on MSC molecular phenotype than donor age [334]. Therefore, to minimize donor-to-donor variations and reduce bioprocessing variability are urgently needed for the production of MSC in large-scale expansion for allogeneic therapies. This undoubtedly emphasizes the importance of harmonizing inter-laboratory practices for manufacturing MSC and products derived from their secretome in order to achieve excellence in the biomedical application of therapy on the basis of these cells.

Conversely, the clinical applications of MSC are attributed to unique stem cell properties, including the secretion of trophic factors involucred in regeneration, as well as pro-angiogenic, anti-inflammatory, immunodulatory or anti-oxidative stress activities. However, there are no acceptable potency assays for the release of MSC for clinical therapies that predict their in vivo efficacy [335]. Thus, the optimal functional characterization of crafted MSC and their secretome should be also necessary, particularly for each specific therapeutic indication.

## 7. Conclusions and Future Perspectives

Stem cell science is a relatively new expertise. Although MSC have long suffered from a relative lack of basic biologic investigations, their conceptualization as a cell-based therapy has evolved from the field of regenerative medicine toward the natural physiological processes. Different paradigms have been constructed to explain their mechanism of action, including tissue regeneration, anti-inflammatory and immunomodulator effects, and anti-cancer and anti-microbial activities. These functions, attributed mainly by the secretion of molecular factors and EVs, have been observed in multiple xenogeneic experimental models, suggesting that their involved mechanisms are conserved between species. These results motivated the empirical clinical use of MSC in multiple clinical trials and recent approved therapies. Nevertheless, it is necessary a better understanding of mechanisms of action and acute and long-term safety profiles, for both the cells and their secretome-derived products.

Heterogeneity of MSC, according their origin, donor characteristics, and in vitro culture conditions, are limitations for clinical applications, but also opportunities for achieving a new efficiently adapted personalized medicine. The most adequate strategies for MSC introduction should be achieved by choosing (i) the most satisfactory MSC type for each therapeutic application, (ii) the most adequate culture conditions for enhancing their specific therapeutic effects, (iii) the most suitable and effective mass production of these cells or derived products by using bioreactors provides of the most highly control parameters, and (iv) the most appropriate functional test for these biological products in each therapeutic indication. For all of these proposes, we need to integrate new technologies, such as related to biotechnology, engineering, and artificial intelligence.

State-of-the-art development in the world of MSC is leading us to the conception of organoid machines that generate signals with medicinal effect. MSC or their derivative products in these systems can contribute to counteract the physiological tissue imbalance after the restoration of truncated homeostasis. In this context, one might consider a different medicine with a certain component of ethical uncertainty. However, we can also conceive of this orientation in the context of an evolution. Humankind has always sought progress in all areas. Our techniques have evolved from warfare to the most advanced military weapons. Yet we can also believe in the therapeutic use of the secretome of MSC in the evolution of medicine towards a sophisticated perception of the molecular balance of life.

## Data Availability

Not applicable.
